# Quantitative ethology of schistosome miracidia characterizes a conserved snail peptide that inhibits host recognition

**DOI:** 10.1371/journal.ppat.1013766

**Published:** 2025-12-09

**Authors:** Rachel V. Horejsi, Chase N. Nelson, Avery De Ruyter, Helen M. Gensch, Saige Maasz-Seawright, Carly Weber, Sophie Willett, Sonja A. Olson, Nicolas J. Wheeler

**Affiliations:** Department of Biology, University of Wisconsin-Eau Claire, Eau Claire, Wisconsin, United States of America; Texas Biomedical Research Institute, UNITED STATES OF AMERICA

## Abstract

Over 700 million people are at risk of contracting schistosomiasis due to regular exposure to freshwater sources where infected snails, the obligate intermediate hosts of schistosomes, are endemic. Although mass drug administration of praziquantel effectively controls the disease in most regions, achieving elimination will require reducing populations of infected snails that shed the human-infective larval stage. Considerable research effort has focused on parasite development and immunological responses after snail penetration, but comparatively little is known about the molecular and behavioral host seeking events that precede it, primarily due to technical and physical constraints. To address this gap, we developed a custom imaging and computational system for tracking and screening schistosome miracidia, the snail-infective larval form that hatches from eggs. Our system employs an array of cameras without magnification and acrylic devices that maintain miracidia within the focal plane, create a field of view over 200,000 times the area of a single miracidium, and support the formation of stable chemical gradients. Using this platform, we perform quantitative ethology of miracidia at an unprecedented scale and extract features that drive the emergent chemoklinokinetic behavior in response to snail cues. We demonstrate that miracidia accumulate at the edge of a gradient of snail cues by increasing key chemoklinokinetic features upon leaving the region of a cue, corroborating previous reports. In contrast, miracidia do not exhibit these behaviors when the cue is uniform, demonstrating that the behaviors represent a specific sensory response rather than generic neuromuscular activity. We further find that a previously identified stimulatory snail peptide only partially recapitulates the full chemoklinokinetic profile, and homologues from closely related snails elicit divergent behavioral outcomes. Notably, some of these snail peptides can mask the natural gradient of snail cues and inhibit miracidia recognition of snails. This work establishes a scalable behavioral platform for probing parasite-snail interactions and identifies a peptide scaffold that potently blocks snail recognition.

## Author summary

Schistosomiasis is a parasitic disease that affects hundreds of millions of people, especially in regions with limited access to clean water. To complete its life cycle, the parasite must infect freshwater snails, a process that begins when its aquatic larvae – called miracidia – locate and recognize suitable snail hosts. In our study, we developed a new imaging and analysis system that allows us to observe and quantify how miracidia behave in response to chemical signals from snails. We found that miracidia change their swimming patterns only when these signals are presented as gradients, not when they are uniformly distributed. We focused on a specific snail-derived molecule, a peptide called P12, and discovered that different versions of this peptide from various snail species trigger distinct behavioral responses. When P12 is spread uniformly in water with a suitable snail host, it can block the parasite’s ability to recognize and infect snails. These findings offer new insight into how parasites locate their hosts and suggest a possible strategy for reducing disease transmission by interfering with host recognition without harming the snails themselves.

## Introduction

Schistosomiasis is a highly prevalent neglected tropical parasitic disease that infects over 250 million people worldwide, with hundreds of millions more at risk. Low-income communities without adequate access to clean water and sanitation are disproportionately impacted [1,2]. Sustained mass drug administration (MDA) of praziquantel (PZQ) has reduced schistosomiasis prevalence and intensity, but because of a lack of control efforts targeting the transmission cycle itself, reinfection and persistent hotspots are common [3,4]. Schistosomes, the parasitic flatworms that cause schistosomiasis, require the progression through a snail intermediate host to become infective to humans, making the snail-infective stage a chokepoint that, when targeted for control, greatly increases the effectiveness of MDA and reduces prevalence even in the absence of MDA [5]. Though much effort has been invested in understanding schistosome development and snail immunological responses following penetration, comparatively little is known about the host-seeking behaviors and molecular events that prelude snail infection.

Miracidia, the free-living, aquatic larvae that hatch from eggs and infect snails, exhibit behavioral alterations in response to chemical signals that indicate a nearby snail host. Most ethological studies of miracidia behavior have relied on qualitative observations that highlight specific features of swimming alterations and accumulation tendencies [6–9]. More recent studies have used video recording and image analysis software to quantitatively describe miracidia behavior [10–13]. From this work, miracidia have been shown to perform chemoklinokinesis when exposed to snail cues [14]. Chemoklinokinesis is a behavioral response in which organisms increase the frequency and magnitude of their turns and the tortuosity of their swimming paths in response to chemical gradients, leading to accumulation in areas of higher concentration. This contrasts with chemotaxis, where organisms exhibit directional movement up a gradient. However, throughout the history of miracidia behavioral parasitology, many reports have been difficult to reproduce due to the variety of experimental approaches and limited rigorous quantitative analyses. For example, some evidence suggests that snail cues do not cause a chemoklinokinetic behavior when presented uniformly, while others have shown the opposite [9,14]. Still other reports suggest that miracidia change their behavior only when moving up a concentration gradient rather than down it [8]. Conflicting reports have led to the preference for assays that measure the emergent accumulation phenotype rather than the behaviors that drive this phenotype [15].

Likewise, little is certain about the specific snail cues that elicit the chemoklinokinetic response in nature, as there are many reports regarding potential bioactive snail molecules [16–19]. Recently a snail-secreted peptide known as P12 was identified and was shown to elicit similar behavioral responses to those caused by snail mucus and snail-conditioned water, suggesting it may play a role in miracidia host-seeking [10]. However, the molecular and ethological details of the role of P12 have not been studied; the schistosome receptor for P12 is not known, and the conservation of P12 in other schistosome vectors has not been explored using recently released snail genomes. Potential diversity in P12 presence and/or sequence could shed light on the different host recognition strategies employed by different schistosome species, and a thorough understanding of the ethological effects of P12 on schistosome miracidia could reveal the necessity or sufficiency of this peptide in host recognition and concomitant infection.

Much of our current understanding of miracidia ethology has been a result of studies evaluating behavior for short time frames and within small fields of view. This limitation is due to physical and technical constraints that make relevant spatiotemporal imaging scales challenging to achieve. Maintaining resolution and magnification within a large enough field of view to accurately capture behavioral profiles of miracidia when they encounter cues is limited by their small size (miracidia are < 200 µm long) and ability to swim in all three dimensions in an aquatic environment. Together, these factors make it difficult to keep miracidia in the focal plane during recording and ensure they are not hidden by shadows. Additionally, the establishment of stable chemical gradients in liquid is difficult to control and reliably reproduce. These limitations have resulted in ethological studies that have often relied on qualitative observations or quantitative measurements limited in scale.

A better understanding of the behaviors involved in host recognition and the host molecules sensed as chemoklinokinetic cues could inform schistosomiasis control efforts. To combat schistosomiasis in regions with persistent hotspots, the World Health Organization recommends snail control through molluscicide treatment or habitat removal [20]. An alternative to widespread extermination of an important ecological regulator may involve reduction in the number of infected snails rather than reduction of the snail population as a whole [21]. Inhibition of miracidia chemoklinokinetic features and the resultant accumulation in snail active spaces may reduce the prevalence of infected snails, either through direct blocking of the chemoklinokinetic process elicited by snail cues, synthetic stimulation of the chemoklinokinetic process to stop miracidia dispersal and entry into the snail active space, or through masking of natural snail gradients.

To explore schistosome miracidia chemoklinokinetic behaviors, define their quantitative features, and investigate behavioral diversity in response to diverse cues, we have developed an imaging platform that employs an array of high-resolution cameras and customizable acrylic arenas that expand the field of view to an unprecedented scale, maintain miracidia within the focal plane, and support stable and reproducible chemical gradients. Using this platform and an expanded quantitative behavioral feature set, we describe the behaviors of miracidia as they explore a large ethology arena with competing chemical cues. To complement the ethology arenas, we have designed a screening arena that allows for high-throughput behavioral screens of miracidia. The combination of these approaches allows us to sensitively describe the chemoklinokinetic features in response to cue gradients or chemical treatments, leading to the identification of molecules that can act on miracidia to block the recognition of snails.

## Results

### High-resolution, wide-field imaging of *Schistosoma mansoni* miracidia in custom arenas enables quantitative description of behavioral features

**Fig 1:**
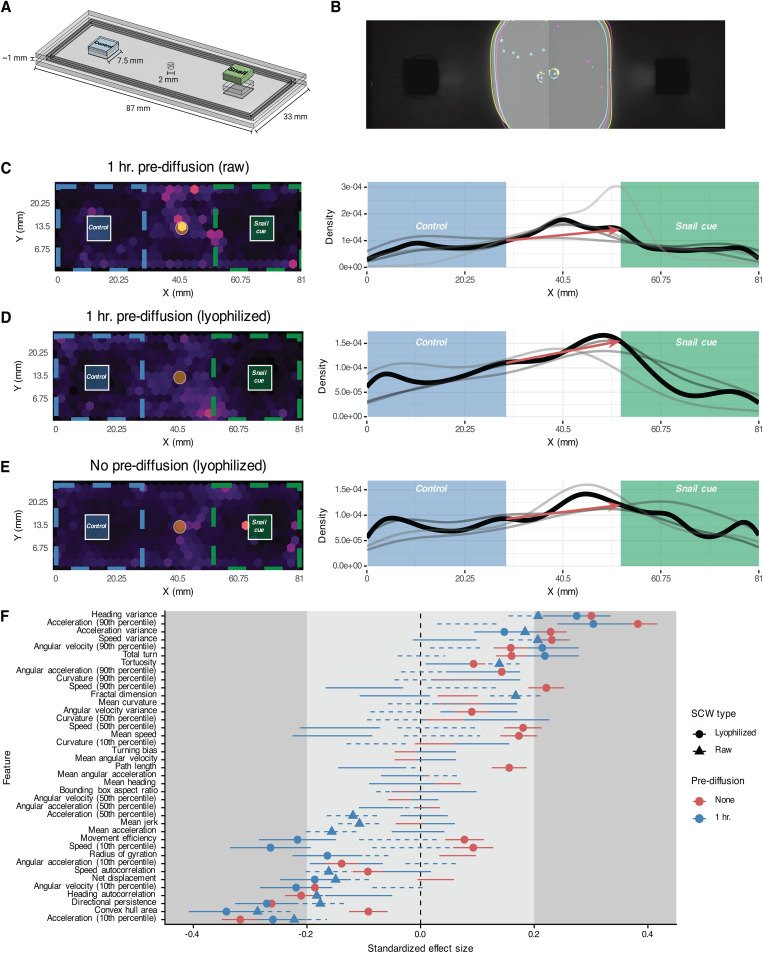
High-resolution tracking of miracidia in a large spatiotemporal scale reveals quantitative ethological features in response to snail cues. (A) Custom choice ethology arena precisely designed to fit the stage of the InVision recording device. Miracidia are loaded through a central pore and allowed to explore the arena. (B) Gradients generated from agarose casts in an ethology arena are highly reproducible. Colored lines represent the gradient boundary after 1 hr. diffusion from three independent replicates using blue dye to track diffusion. (C–E) Left: Heatmaps represent track density over 1 hr. long videos. Right: Density of tracks along the x-axis. Thin grey lines represent independent replicates, and the dark black line represents the mean density across all replicates. Red arrows show the difference in densities at the edge of the boundaries. All three panels include data from >3 biological replicates (miracidia cohorts from independent infections). (F) Quantitative features extracted from tracks in the cue or control regions were modeled with linear mixed-effects models. Standardized effect size is Cohen’s D; lines represent the standard deviation of the estimate. Small, medium, and large effect sizes are distinguished by gray boxes. Only lines with points are statistically significant (FDR < 0.05). Positive effect sizes indicate increase in the feature in the cue region when compared to the control region.

The behavioral and molecular processes involved in schistosome miracidia host-seeking of snails has long been of interest but has been limited by technical constraints that inhibit high-resolution tracking of miracidia at relevant spatiotemporal scales. We overcame these constraints through design and fabrication of a custom imaging device combined with bespoke “ethology arenas” produced in-house (Figs 1A, 1B and S1). Our InVision (for invertebrate vision) is inspired by a similar solution for wide-field simultaneous imaging of *C. elegans* in large phenotypic screens but leverages a unique arena design customized for aquatic organisms [22,23]. The InVision deploys a 2x2 array of high-resolution (126.5 px/mm at 20 cm working distance) cameras, utilized in pairs to image two ~ 9x3 cm acrylic arenas (Fig 1A). Arenas are laser-cut and adhered with acrylic solvent, with a thin frame separating the top and bottom layers to limit the Z-axis movement of miracidia and ensure consistent localization within the camera’s focal plane (representative videos of miracidia in a custom arena can be found at BioImage Archive S-BIAD2286). Cut slots in the top layer enable the positioning of agarose casts made with cues of interest. Combination of acrylic arenas and agarose casts produced stable and reproducible gradients after 1 hour of diffusion, which was optimized using dye and recording its diffusion in the arenas over time (Fig 1B and S1 Movie). In three independent diffusion optimization runs, the edge of each gradient overlapped, showing remarkable consistency in gradient formation (Fig 1B).

To validate our imaging platform, miracidia behavior was recorded in choice ethology arenas using snail-conditioned water (SCW) as a cue, which has been shown to cause behavioral changes and accumulation when experienced in a gradient [8,15,18]. We used raw, unconcentrated SCW and lyophilized, concentrated SCW to test if SCW concentration influenced miracidia behavior in our experimental setup. We used lyophilized SCW in experiments with and without pre-diffusion of cue. Without pre-diffusion, the concentration gradients were consistently and dynamically expanding during the experiment, while with 1 hr. pre-diffusion the gradients stabilized, creating a sharp border between cue, control, and middle regions.

With pre-diffusion of either raw or lyophilized SCW, parasites accumulated along the border of the SCW region, with the starkest difference occurring when using lyophilized SCW, which had the strongest and sharpest gradient (Fig 1C–1D). Miracidia also accumulated nearer the SCW region when the cue had not been pre-diffused, but the border accumulation was less pronounced (Fig 1E). Interestingly, in no experiment did miracidia accumulate next to the cue source, which is consistent with the steep gradient supported by our arenas and the “boundary reaction” described in classical ethological studies, which maintains miracidia in the active space of a snail [8,24].

A key advance in our wide-field tracking approach is the ability to extract quantitative behavioral features that describe the kinematics, path geometries, and spatial and angular dynamics of miracidia tracks. The combination of these features creates a “behavioral profile,” an aggregate pattern formed by multiple quantitative features measured across track segments. While any single feature may or may not be different between experimental conditions, it is the combination of features that provides a robust and nuanced characterization of biological behavior.

To systematically compare these behavioral profiles, we computed a set of 42 quantitative features from short track segments (“chunks”) and modeled the behavioral responses with linear mixed-effects models (LMMs), which account for within-video and within-individual variation. Because we track miracidia in the entire arena and maintain stable, non-overlapping gradients, we can compare behavioral features in different regions of interest using LMMs. After correcting for multiple comparisons, we found many behavioral features that were significantly different between cue and control regions for experiments with pre-diffusion (lyophilized: 13 features, raw: 15 features, FDR < 0.05) and without pre-diffusion (24 features) (Fig 1F). In all experiments, heading variance and acceleration (90^th^ percentile) had the greatest positive effects, while directional persistence, convex hull area, and acceleration (10^th^ percentile) had the greatest negative effect. Overall, the behavioral profiles were remarkably similar between experiments, with only two features showing opposite effects. Interestingly, where significantly different features showed the same directional effect in all experiments, the miracidia in arenas without pre-diffusion routinely exhibited greater changes in behavior (as measured through absolute value of standardized effect size), which may be explained by the dynamic gradient requiring a more refined and sensitive response (Fig 1F).

**Fig 2:**
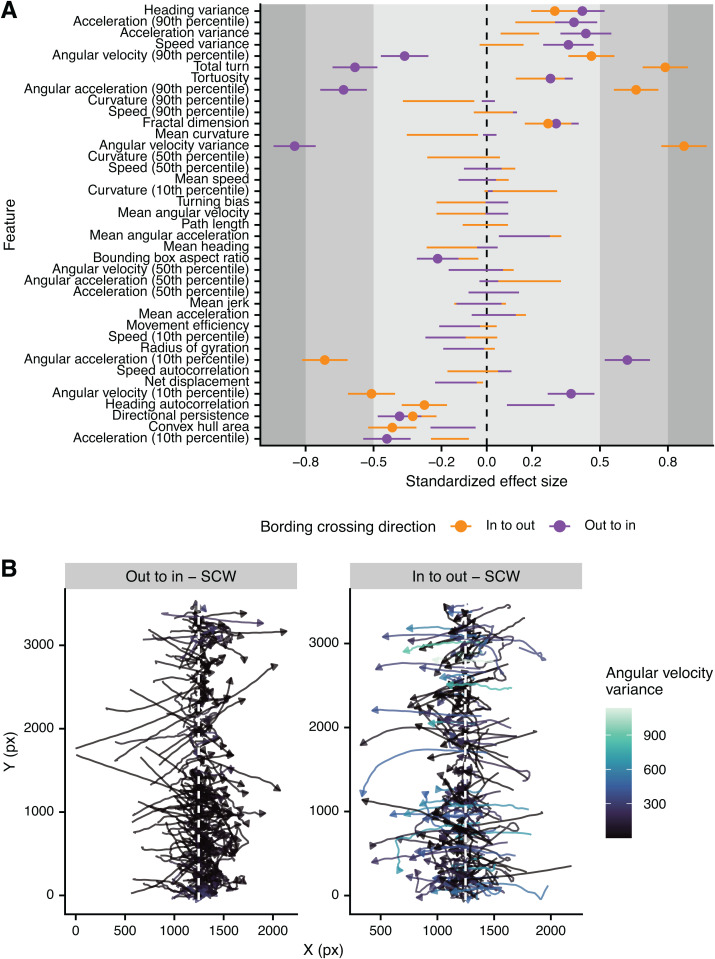
Quantitative description of the boundary reaction of miracidia moving in or out of a cue region. (A) Quantitative features were extracted from track chunks crossing the boundary of cue or control regions and modeled as a response of region. Standardized effect size is Cohen’s D; lines represent the standard deviation of the estimate. Small, medium, and large effect sizes are distinguished by gray boxes. Only lines with points are statistically significant (FDR < 0.05). Positive effect sizes indicate increase in the feature when crossing the cue boundary when compared to the control boundary. Features on the y-axis are in the same order as Fig 1F. (B) Extracted boundary crossing track chunks showing the increased turning of miracidia moving out of the cue region in comparison to those moving into the region.

In contrast to the few quantitative behavioral features that have been analyzed in the past, accumulation at a gradient has been a consistent observation in descriptive studies of miracidia behavior. However, how this accumulation is accomplished has not been made clear. Some reports suggest that moving up the gradient (i.e., “out to in”) does not lead to a behavioral change; instead, moving down the gradient (i.e., “in to out”) will lead to behavioral changes that result in accumulation and maintenance within the active space of the cue [8,25]. Others describe behavioral changes when moving in either direction [18,26]. Our choice ethology arenas allow clear comparisons between the behaviors exhibited while moving in to or out of a cue region. When comparing only track chunks that crossed a cue or control boundary in pre-diffused experiments (i.e., tracks moving into the cue region in comparison to tracks moving into the control region), we found more behavioral features that were significantly different for tracks moving out of a cue region (15 features) than into a cue region (11 features) (Fig 2A). Remarkably, while the overall profile was similar for the two behaviors, there were six features that were affected in the opposite direction and were greatly dissimilar – miracidia moving out of the cue region (“in to out”) had more turns and a higher variance in the angular velocity (Fig 2B). These differences support the previously described “boundary reaction”, which ensures that miracidia do not leave an active space after serendipitously discovering it [8,24]. Our data suggests that miracidia perform small to moderate changes in behavior when experiencing either environmental change – moving into or out of a cue – but will substantially increase their turning when leaving a cue.

### Natural variation in a conserved *Biomphalaria* peptide causes divergent behavior and accumulation effects on *S. mansoni* miracidia

**Fig 3:**
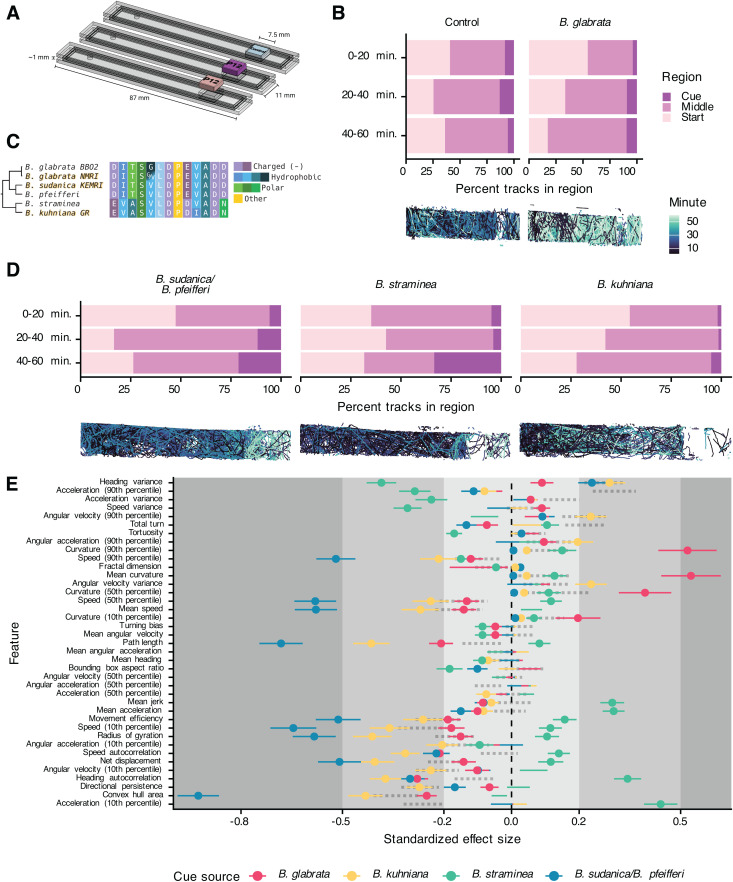
Single-cue ethology arenas allow side-by-side comparisons of the quantitative behavioral features in response to *Biomphalaria* spp. P12 peptides. (A) Custom single-cue ethology arenas allow for simultaneous imaging of three arenas per InVision camera pair. (B) Top: Number of tracks per region (1/3 of the arena) over time. Bottom: Representative tracks. Miracidia progressively accumulate nearer the BgP12 cue but not the control cue. (C) Multiple sequence alignment of cloned and reference P12 homologs. Gold-shadowed sequences were cloned. Cladogram derived from [29]. (D) Same as (B) but with new *Biomphalaria* homologs. (E) Extracted behavioral features from track chunks in the cue region were compared to control experiments. Standardized effect size is Cohen’s D; lines represent the standard deviation of the estimate. Small, medium, and large effect sizes are distinguished by gray boxes. Only lines with points are statistically significant (FDR < 0.05). Positive effect sizes indicate increase in the feature in the cue region when compared to the control region. Features on the y-axis are in the same order as Fig 1F; dotted lines represent the values from lyophilized SCW in Fig 1F.

Our novel platform and initial experiments with SCW began to unravel the quantitative ethology of the miracidia response to snail cues, and we next moved to exploring the role of specific snail cues that may be involved in eliciting the miracidia response. Much effort has been given to identifying molecules within SCW that contribute to the behavioral effect, with data suggesting that glycoproteins, salts, or even protons could be involved [9,18,27,28]. Recently, a secreted snail peptide (P12) was identified in fractionated *B. glabrata* SCW and shown to cause accumulation and behavioral change when miracidia were exposed to it in a gradient [10,11,19]. To allow side-by-side comparisons of multiple cue sources rather than a binary choice between two cues, we designed single-cue ethology arenas to simultaneously record miracidia from the same cohort responding to different cues (Fig 3A). Like the choice ethology arenas, we verified that stable and reproducible gradients could be produced (S2 Movie).

We initially tested these arenas by comparing the response of miracidia to a gradient of BgP12. When compared to the control, miracidia progressively accumulated in regions nearer the BgP12 source over time (Fig 3B). Behavioral features of chemoklinokinetic responses were significantly altered when miracidia were in the presence of BgP12. Although the behavioral profile was similar to that caused by lyophilized SCW, several features were markedly different (Fig 3E).

At the time of discovery, BgP12 was not known to have homologs in other snails due to a lack of gastropod genomic resources. Using the *B. glabrata* BB02 reference sequence for P12 [30], we found homologs in *B. pfeifferi* [31], *B. straminea* [32], and *B. sudanica* 111 [33] reference genomes. Notably, we did not find a P12 homolog in the *Bulinus truncatus* genome [34], which is the primary African vector for *Schistosoma haematobium* and a planorbid related to *Biomphalaria*, or the genome of *Oncomelania hupensis*, the vector of *Schistosoma japonicum* and not closely related to the planorbid schistosome vectors [35]. Thus, P12 seems to be restricted to *Biomphalaria*. Using primers designed using the full-length mRNA that encodes the putative BgP12 pro-peptide, we cloned sequences from *B. glabrata* NMRI, *B. sudanica* KEMRI, and *B. kuhniana* GR [36] (GenBank accession numbers: PV848035- PV848039). Amongst these five related *Biomphalaria* snail species, four versions of the P12 peptide were identified and varied at seven residues, as the P12 from the closely related *B. pfeifferi* and *B. sudanica* were identical. Our lab population of *B. glabrata* NMRI was found to be heterozygous at one position in the P12 peptide sequence (Fig 3C).

We synthesized these peptides and used them in single-cue ethology assays. The *B. sudanica/B. pfeifferi* and *B. straminea* sequences caused clear accumulation over the length of the experiment, while the *B. kuhniana* peptide had a moderate effect similar to the control (Fig 3D). These results suggest that accumulation is not a response to a general peptide gradient but is specific to the P12 sequences. These data partially correlate with what is known about compatibility between *S. mansoni* and *Biomphalaria* spp.: *S. mansoni* NMRI readily infects *B. glabrata, B. pfeifferi, B. sudanica,* and *B. straminea* but does not infect *B. kuhniana* [36].

We extracted features from tracks and compared differences between tracks on the cue-half or entry-half of the arenas. There was not a generic behavioral profile in response to P12s, and profiles varied from the lyophilized SCW profile (Fig 3E). Instead, the *B. sudanica/B. pfeifferi* and *B. straminea* peptides, which caused the greatest amount of accumulation near the cue, caused very different behavioral features but consistently resulted in higher effect sizes than the *B. glabrata* or *B. kuhniana* homologs. Even though *B. kuhniana* did not cause strong accumulation, it still caused some behavioral change, but most features differences were of small effects (Fig 3E).

These data indicated that all four *Biomphalaria* P12 variants elicit chemoklinokinetic behavioral features but different behavioral profiles. Miracidia accumulation nearer the cue region also differed, with the *B. sudanica/B. pfeifferi* P12 causing the most accumulation while also leading to the greatest effects on chemoklinokinetic features. Since accumulation is an emergent property of chemoklinokinetic features, it is notable that the cues that caused the greatest accumulation also had largest effects on quantitative features.

### Snail-derived cues only cause miracidia chemoklinokinesis when presented in a gradient

**Fig 4:**
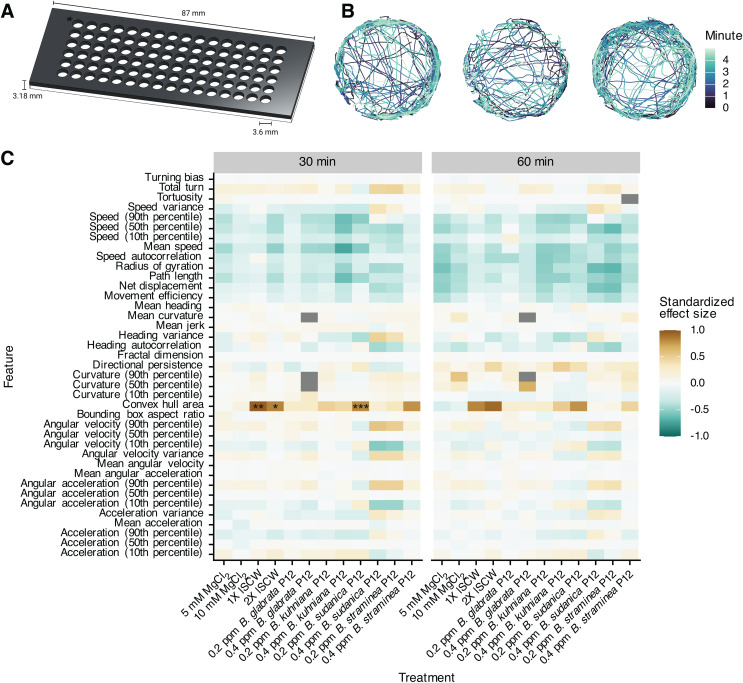
High-throughput behavioral screening of chemoklinokinetic stimulants. (A) Custom screening arena for simultaneous recording of 96 wells per InVision camera pair. (B) Representative tracks of three wells. (C) Extracted behavioral features from track chunks in the treated wells were compared to control wells. Standardized effect size is Cohen’s D. Positive effect sizes indicate increase in the feature in the treated wells when compared to the control wells. P-values are adjusted with Holm’s correction (*p ≤ 0.05; **p ≤ 0.01, ***p ≤ 0.001).

Based on data showing that P12 homologs cause accumulation and diverse behavioral effects, we reasoned that P12 may be a potential chemoklinokinetic stimulant that could block snail infection by inhibiting the ability of miracidia to accumulate in the active space of snails. To determine an effective concentration for P12 exposure, we designed a high-throughput behavioral assay that would enable dose-response experiments. Our custom screening arena maintains the advances of the ethology arenas (no meniscus, limited Z-axis movement) while allowing 96-wells to be simultaneously recorded by each camera pair (Fig 4A). Tracking miracidia over 5 minute videos results in high-quality, contiguous paths (Fig 4B).

We exposed miracidia to P12 at concentrations consistent with those experienced in ethology arenas. As potential positive controls, we also used lyophilized SCW and MgCl_2_, which has previously been shown to drive chemoklinokinetic behavior and accumulation [16,37]. We recorded miracidia immediately after exposure and every 30 minutes for 90 minutes. After 30 or 60 minutes, most quantitative features were not significantly different from negative control wells, even treatments used as positive controls (Fig 4C). There were some differences immediately after exposure, but the effect sizes were small and were lost by 30 minutes (S2 Fig). We reproduced these results with SCW and MgCl_2_ using droplets on a slide to ensure that negative results were not an artifact of our screening arena [38] (S3 Fig). These data show that the behavioral changes exhibited in ethology arenas are dependent upon a gradient – uniform presentation of the cue does not cause chemoklinokinesis. The changes caused by SCW and P12 peptides are thus truly sensory in nature and not a generic neuromuscular stimulation, as the gradient requirement has been repeatedly shown in other metazoan systems that use chemotaxis [39–41]. Notably, the contrast in miracidia behavior when in a gradient or in a uniform concentration has been a topic of some disagreement in the literature [42].

### Uniform presentation of miracidia to *Biomphalaria* P12s inhibits recognition of snails

**Fig 5:**
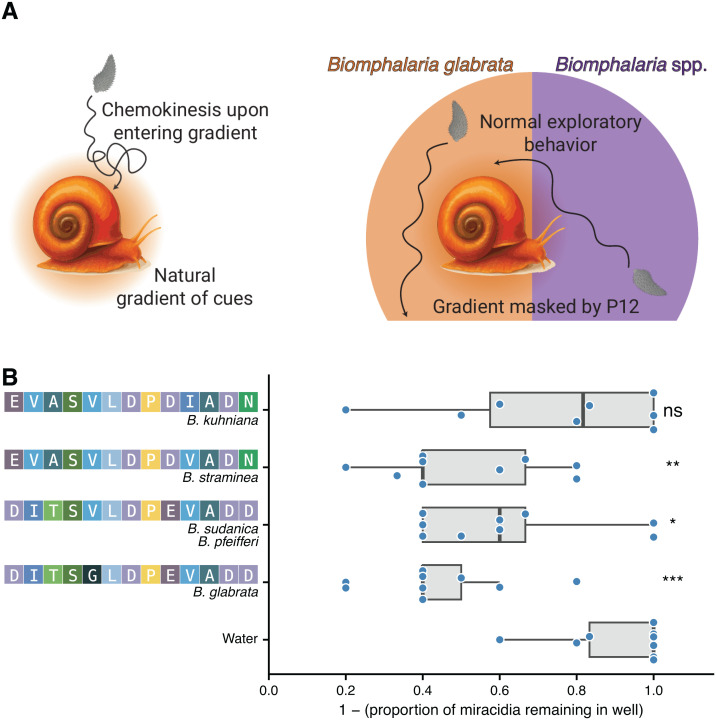
Inhibition of snail recognition by gradient masking. (A) Theoretical model for how *Biomphalaria* P12s may block miracidia recognition of snails. (B) Fraction of miracidia remaining in well after 1 hour incubation with snails in the presence or absence of *Biomphalaria* P12 peptides. Each point represents an independent experiment (snail in a well with miracidia). Data is the combination of three biological replicates (three snails exposed to miracidia from independent infection cohorts). Adjusted p-values are the result of Dunnett’s test (*p ≤ 0.05; **p ≤ 0.01, ***p ≤ 0.001).

The inability to activate chemoklinokinesis in high-throughput context impeded our ability to identify effective concentrations that would act as chemoklinokinetic stimulant to block dispersal in a natural environment. However, these results led us to reason that P12 peptides may be able to mask the natural snail cue gradient and inhibit the behavioral processes that lead to recognition and infection. Miracidia exhibit chemoklinokinetic responses when they enter the active space created by natural snail secretions. In this theoretical framework, a masked gradient would cause miracidia to exhibit normal exploratory behavior instead of chemoklinokinesis, even after having entered a snail active space (Fig 5A).

To test our hypothesis, we conducted host recognition experiments with miracidia and snails in the presence or absence of P12 peptides. After incubating miracidia with snails for 1 hour, 90% of untreated miracidia were no longer in the well after removing the snail, suggesting that they had successfully initiated the infection process by recognizing and penetrating the snail. When we added *Biomphalaria* P12s, the peptides from *B. glabrata, B. straminea,* and *B. sudanica/B. pfeifferi* significantly increased the number of miracidia that remained in the well after 1 hour (Fig 5B). *B. glabrata* P12 was the most effective, causing a > 50% increase in the number of miracidia left behind after removing the snail. Remarkably, *B. kuhniana* P12, which differs from *B. straminea* P12 by only a single amino acid and *B. glabrata* P12 by 5 amino acids, did not have a significant effect on host recognition (Fig 5B). These data are consistent with the ethology experiments, which showed that BkP12 caused less accumulation than the other P12s and had smaller effects on quantitative behavioral features (Fig 3).

## Discussion

We developed a scalable imaging and analytical platform that enables high-resolution, quantitative ethology of schistosome miracidia. This approach captures their behavior at previously inaccessible spatiotemporal scales and helps reconcile decades of fragmented observations in behavioral parasitology.

Our platform allowed us to explore miracidia behavior in response to a secreted *Biomphalaria* peptide – P12 – that was identified by screening *B. glabrata*-conditioned water fractions for their ability to induce chemoklinokinesis in *S. mansoni* miracidia [10]. Sequence homology searches found P12 homologs only in *Biomphalaria* spp., not in *Oncomelania* or *Bulinus*, the primary intermediate hosts for *S. japonicum* and *S. haematobium*, respectively. This raises the possibility that alternative cues are used by these two species. Intriguingly, *S. japonicum* miracidia perform directed chemotaxis up a concentration gradient of cues from *B. glabrata*, even though it is not their natural host [59]. Testing for P12’s effect on *S. japonicum* and *S. haematobium* could clarify whether the P12 receptor and its downstream signaling pathway are shared in schistosomes, and, if so, whether this pathway is used for divergent behaviors.

The divergent behaviors in *S. mansoni* caused by sequence diversity among *Biomphalaria* P12s may be correlated with the history of *S. mansoni* host range expansion. *S. mansoni* likely originated in East Africa, where it used *B. pfeifferi, B. sudanica, and B. alexandrina* as hosts but expanded to additional *Biomphalaria* species as it spread across the continent [43]. *S. mansoni* colonization and establishment in the Neotropics (primarily via *B. glabrata* and *B. straminea*) is a relatively recent event, and it is likely that *S. mansoni* was pre-adapted to infect these species [44]. Previous studies on *S. mansoni* recognition of SCW from different snails showed that African populations were more responsive to SCW from African snails, while Neotropical populations did not differentiate among SCW, even from incompatible snail hosts [45,46]. It is not known whether the population used here, *S. mansoni* NMRI, which was originally collected from Puerto Rico but has been maintained in the lab for more than 80 years, can differentiate between snail species [47]. However, our data show divergent behavioral effects in response to small changes in the P12 sequence. Site-directed mutagenesis of the 13 P12 residues could reveal key amino acids required to maintain bioactivity, which could help generate a scaffold for the design of peptidomimetics or macrocycles for use as infection blockers [48].

While our study identifies behavioral signatures linked to successful host recognition and further implicates conserved *Biomphalaria* P12 peptides as modulators of host-finding, their molecular targets in miracidia remain unknown. Uncovering how miracidia detect these cues may require integration of single-cell transcriptomics, peptide-receptor mining, and new functional tools [49–51]. Although reverse genetics in larval schistosomes remains technically challenging, our platform offers a robust foundation for bridging behavior with molecular mechanisms in this host-parasite interface.

Beyond basic discovery, our experimental and computational capabilities support translational research by enabling the identification and characterization of compounds that stimulate or block chemoklinokinetic responses. Either approach may effectively block snail infection, which would be a major advance and an additional tool for schistosomiasis control. Given the ecological importance of aquatic snails, our strategy for reducing the number of infected snails rather than the general snail population may offer a preferable alternative for control approaches targeting the snail stages. Quantitative ethology of miracidia in response to prioritized compounds may help predict effective infection blockers.

There remains opportunity for further advancement. Our finding that snail cues fail to induce chemoklinokinesis under uniform presentation poses a challenge for high-throughput screening. Effective inhibitor screening requires consistent, reproducible activation of behavior, which is currently limited by the need for stable gradients. It remains unclear whether this limitation applies only to sensory-level stimuli or also to downstream neuromodulators. Compounds that act beyond the initial sensory detection may still prove effective. To overcome this, future screens could leverage microfluidic technologies that support miniaturized gradient formation.

If miniaturization of a behavioral screening platform is successful, the identification of chemoklinokinetic stimulants or blockers would be enhanced by further scrutinizing the quantitative features extracted from tracks, with the goal of creating non-redundant behavioral fingerprints. Here, we compared individual features and described the profiles created by all 42 features. However, correlation among some of these features may be high, and some features may be more biologically meaningful than others. The selection of uncorrelated, meaningful features, followed by mathematical clustering of profiles, could reveal fingerprints that can be associated with qualitative ethograms or used to identify bioactive molecules in screening approaches [52,53].

## Materials and methods

### Ethics statement

Livers from *S. mansoni* (NMRI)-infected female Swiss outbred mice (The Jackson Laboratory) were harvested at the Biomedical Research Institute (BRI) at 6 weeks post-infection and shipped overnight in perfusion fluid to UWEC. BRI is a USDA Registered Research Facility (#51-R-0050), an AAALAC accredited facility, and has an institutional PHS Assurance (#3080-01) from the Office of Laboratory Animal Welfare (OLAW): D16-00046 (A3080-01).

### Snail husbandry and parasite maintenance and harvesting

*Biomphalaria glabrata* (NMRI population) was maintained in two separate aquaria: a 30-gallon main colony tank and a 20-gallon nursery for eggs and juvenile snails. Both tanks were filled with artificial pond water (APW) and maintained at 25°C with constant aeration. Snails were fed three times per week with one large leaf of organic romaine lettuce per tank and supplemented with a small amount of fish food (Tetramin). Egg masses were deposited on floating pieces of Styrofoam. Styrofoam pieces with high densities of viable egg sacs were transferred to the nursery tank for hatching. Juveniles were returned to the main tank once sufficiently grown. Snails are unregulated animals and do not require ethics approval.

*S. mansoni* m*iracidia* were harvested as previously described [54,55]. Miracidia density was calculated by counting five 10 µL aliquots stained with 1:1 Lugol’s iodine and diluted with fresh APW to a final density of 1 parasite/µL to use in ethology assays and variable final densities for high-throughput screening assays. Miracidia were directly transferred from the harvesting flask to a glass petri dish and individually pipetted for host recognition experiments.

### InVision, arena and agarose mold design and fabrication

Detailed methods for design and fabrication of the imaging system and its components can be found in S1 Methods. For high-resolution wide-field imaging, we designed the InVision (for invertebrate vision) by customizing a Kastl-HighRes unit controlled by the Motif software (Loopbio GmbH, Vienna, Austria) based on inspiration from similar recording devices for *C. elegans* [22] (S1 Fig). The enclosure is not environmentally controlled but has two exhaust fans to maintain ambient temperature. Behavioral arenas sit upon a stage with a 200 x 200 mm white LED panel (MBJ DBL-2020 series, Ahrensburg, Germany) below to generate a homogenous bright background by transillumination. Four cameras in a 2x2 array are paired to create two distinct fields of view (FoV) per arena, covering 81x27 mm or an entire single arena.

Design files (PDF, STL, or CAD) for behavioral arenas and molds can be found in GitHub repository associated with this manuscript (wheelerlab-uwec/miracidia-sensation-ms) or the archived release on Zenodo (10.5281/zenodo.17253221). All arena parts were fabricated from laser-cut 1.6 mm (1/16”) thick clear or black cast acrylic (McMaster-Carr, Elmhurst, IL USA). Parts were custom designed to perfectly fit the InVision stage, each arena being comprised of a layer of acrylic affixed with an acrylic solvent. For choice and single-cue ethology arenas, three clear pieces were cut: a base with an engraved groove for seating the frame, a frame, and a top with engraved groove and cut inlets for loading agarose casts or parasites. Parasites are added to a central pore for choice arenas and allowed to explore cue/control gradients emitting from agarose casts on each end of the arena. Parasites are added to one end of the arena for single-cue arenas and allowed to explore a single cue/control gradient. For screening arenas, a single clear base was cut, and a black top was cut with wells designed to fit a multichannel pipette. All arenas limit miracidia movement in the Z-axis, maintaining the parasites within the cameras’ focal plane.

Agarose molds were designed to create casts that perfectly fit the choice and single-cue ethology arenas and hold 180–200 µL of liquid.

### Miracidia choice and single-cue ethology assays

Snail-conditioned water (SCW) was prepared by incubating 20 *B. glabrata* NMRI snails (8–15 mm) in 25 mL of APW in a glass beaker for 3 hours at 28°C [10]. 2.5 mL aliquots of the resulting solution were lyophilized (Labconco FreeZone, Kansas City, MO USA), and the resulting powder was stored at -80°C. All experiments were performed with aliquots from the same batch of lyophilized SCW.

Agarose casts of cues were made by making 0.5% agarose in APW and pipetting molten agarose into 3D-printed agarose molds. The molds were placed in a humid box and kept in the refrigerator for 12–15 minutes to polymerize. For diffusion tests used to optimize the system and image the diffusion of a chemical over time, 1% Brilliant Blue dye was added to the 0.5% agarose casts. For the choice ethology assays, lyophilized SCW agarose casts were made by adding approximately 180–200 µL of 0.5% agarose solution to a 2.5 mL lyophilized SCW aliquot, mixing the components via pipetting, and adding the mixed solution to the agarose molds. A single 2.5 mL aliquot consists of the combined secretory products from ~2 snails over 3 hrs. APW agarose casts were also made and used in the choice ethology assays. For the single-cue ethology assays, 2X stocks of each synthesized *Biomphalaria* P12 peptide was made by diluting 2 mg aliquots of the peptides with 1 mL APW. 1 mL of the 2X stocks was mixed with 1 mL of 1% agarose solution, for a final concentration of 1 mg/mL in 0.5% agarose, which was then pipetted into molds.

To prepare for imaging, arenas were wiped down with 70% ethanol and filled with ~3 mL APW in choice ethology arenas and ~1 mL APW in single-cue ethology arenas. Agarose casts were added to the filled arenas and incubated at room temperature for 1 hour to form a cue gradient before adding miracidia. 20 µL of miracidia (1–3 hours old) at 1 parasite/µL were added to the entry pores of the arena(s) and placed in InVision for recording. Arenas were transferred to the InVision and recorded for 1 hour at 8 frames per second (FPS). For each treatment condition (raw SCW, lyophilized SCW with or without pre-diffusion), three independent biological replicates were conducted using miracidia from separate infections.

### High-throughput phenotyping of miracidia

High-throughput behavioral phenotyping was performed with screening arenas and the InVision device. Treatments included four P12 peptides at final concentrations of 200 ng/mL and 400 ng/mL, magnesium chloride at 0.5 mM and 1 mM, and reconstituted lyophilized SCW at volumes of 2 µL and 4 µL per well. Each 10X treatment was prepared in PCR strip tubes, and 1.6 µL of each solution was pipetted into each well of the screening arena using a multichannel pipette (except for SCW, which was pipetted as 2 µL or 4 µL using a single channel pipette). Miracidia were collected after 15 minutes of light induced hatching and suspended in APW at 1 parasite per µL. The miracidia solution was prepared in a 5 mL reservoir with a total volume of 1,500 µL to ensure all 78 wells could be filled. After loading the treatments, 14.4 µL of the miracidia solution was added to each well using a multichannel pipette (12 µL and 14 µL for the 4 µL and 2 µL SCW wells, respectively, to maintain a total volume of 16 µL per well).

For each treatment condition, six technical replicates (wells) were performed per arena, and three independent biological replicates were conducted using miracidia from separate infections. The screening arena was marked in the top left corner to maintain consistent orientation and placed in the InVision system for behavioral recording. Videos were recorded for 5 minutes at 0 (immediately after miracidia were added to pre-loaded wells), 30, 60, 90, and 120 minutes post-treatment. Between recordings, the arena was incubated at 26°C in a sealed chamber with a water reservoir to minimize evaporation.

### Computational tracking and statistical analysis of miracidia in wide-field InVision videos

All analytical scripts and Snakemake workflows are available in the invision-tools GitHub repository (wheelerlab-uwec/invision-tools). An archived release of the code used for this manuscript is on Zenodo (10.5281/zenodo.17095614). Videos from each camera in the 2x2 array were tracked independently and merged afterward if needed. Computational tracking of miracidia is split into two steps, each performed with functions from the soft-matter/trackpy v0.6.4 library [56]: segmentation and linking. For segmenting miracidia, the background was generated every 25 frames by taking the maximum projection of a 25-frame chunk. This background was subtracted from each frame in chunk, and tp.batch was used to identify objects in the foreground. Object coordinate data was stored in an HDF5 file, which was then amended by the linking step performed by tp. For videos containing multiple units (single-cue ethology arenas with 3 units per video or screening arenas with 48 units per video), tracks were split with a custom Python script. For ethology arenas that spanned two cameras, tracks from camera pairs were linked to create a single combined pickle file. All tracking workflows were managed by a Snakefile with a modified Slurm [57,58]. Tracking analyses were performed on the BOSE cluster in the Blugold Center for High Performance Computing at UWEC.

Tracking data can be found on Zenodo (10.5281/zenodo.15732765). Analytical code can be found in the GitHub repository associated with this manuscript (wheelerlab-uwec/miracidia-sensation-ms) or the archived release on Zenodo (10.5281/zenodo.17253221). Tracks of miracidia had sporadic gaps resulting from miracidia interactions with the walls of the arena or agarose casts. To account for the wide range of track lengths, only tracks that consisted of >40 frames (~5 seconds) were used for analyses. Furthermore, each track was split into 5 second chunks and analyzed individually. This chunking scheme ensured that behavioral differences were not artifacts of differences in track length distributions between videos or arena regions and to ensure that long contiguous tracks were properly represented in the final dataset.

Forty-two kinematic, geometric, path, and spatial were extracted from each 5 second chunk (S1 Table). Each behavioral feature was modeled with linear mixed-effects models (feature ~ response + (1 | date/video/track) [59–61] where the fixed response variable was a region (for choice or single-cue ethology arenas) or a treatment (for screening arenas). The model controls for random variation among miracidia extractions and accounts for the nested data structure where 5-second chunks are nested within tracks, tracks within videos, and videos within extraction dates. This approach corrects for the non-independence of multiple chunks from the same track and prevents artificial inflation of sample size by repeated measures. Effect sizes were calculated using Cohen’s D, employing the pooled standard deviation for each feature [62]. Significant differences in behaviors in ethology arenas were assessed based on a false discovery rate (FDR, Benjamini-Hochberg procedure) threshold of <0.05, and multiple testing in screening arenas was accounted for with the Holm’s correction [63,64].

### P12 cloning, sequencing, and synthesis

Predicted sequences of BGLBO28940 and BGLBO27975 were obtained from the *B. glabrata* BB02 reference genome [30], available on VectorBase [65], and Primer3 [66] was used to design PCR primers that would amplify the full-length mRNA and the coding sequence of both genes.

Detailed methods for P12 cloning can be found in S1 Methods. The predicted protein sequences were used in tblastn search to identify potential homologs in other snail intermediate hosts. Homologs *B. glabrata* (NMRI), *B. sudanica* KEMRI, and *B. kuhniana* Grande Riviere [36] were cloned and sequenced (GenBank accessions PV848035-PV848039). Validated sequences were synthesized for use in ethology assays and host recognition experiments (GenScript, Piscataway, New Jersey).

### Snail recognition experiments

Wells of a 24-well plate were filled with 1.2 mL of APW, and 5–6 miracidia were individually pipetted into each. After miracidia were added, the peptides were diluted to 1 mg/mL and added to their respective wells for a final concentration of 0.4 mg/mL. An equivalent volume of APW was added to the control well. Lastly, a single *B. glabrata* snail (6–10 mm) was added to each well and the plate was left to incubate at room temperature for 1 hour. After incubation, snails were removed, and the remaining miracidia were stained with Lugol’s solution and counted. Three biological replicates were performed (independent miracidia harvests from different infection cohorts), each with three snails. We applied Dunnett’s test to compare each treatment group to the water control. Before performing the test, we assessed normality by fitting a linear model and inspecting QQ plots of the residuals for each treatment group.

## Supporting information

S1 Fig Photo of the custom InVision device, which includes a 2x2 array of cameras, an LED back light, and a stage that holds 2–4 arenas per experiment. (JPEG)

S2 Fig Data for high-throughput behavioral screen after 0 and 90 minutes. Data for 30 and 60 minutes is shown in Fig 4.
(PDF)

S3 Fig Behavioral screen of cues from Figs 4 and S2 with miracidia in droplets on slides instead of a screening arena. (PDF)

S1 Movie Representative animation depicting the diffusion of dye in a choice ethology arena every 10 minutes for over the span of 3 hours. (GIF)

S2 Movie Representative animation depicting the diffusion of dye in an ethology arenas every 10 minutes for over the span of 3 hours. (GIF)

S1 Table Mathematical and biological descriptions and interpretations for quantitative ethological features. Mathematical expression column describes programmatic formulas used to compute each feature. Mathematical description column presents verbose explanations of what is being computed. Behavioral description column describes features in an ethological context. Value interpretation column presents what high and low and/or positive and negative values indicate.
(XLSX)

S1 Methods Complete methods for InVision design; arena and agarose mold design/fabrication; and P12 cloning, sequencing, and synthesis. (DOCX)
