# Supplementary figures and images for: Quantitative ethology of schistosome miracidia characterizes a conserved snail peptide that inhibits host recognition
Source: PLoS Pathog. 2025 Dec 9;21(12):e1013766. doi: 10.1371/journal.ppat.1013766 (PMC12704881; doi:10.1371/journal.ppat.1013766)

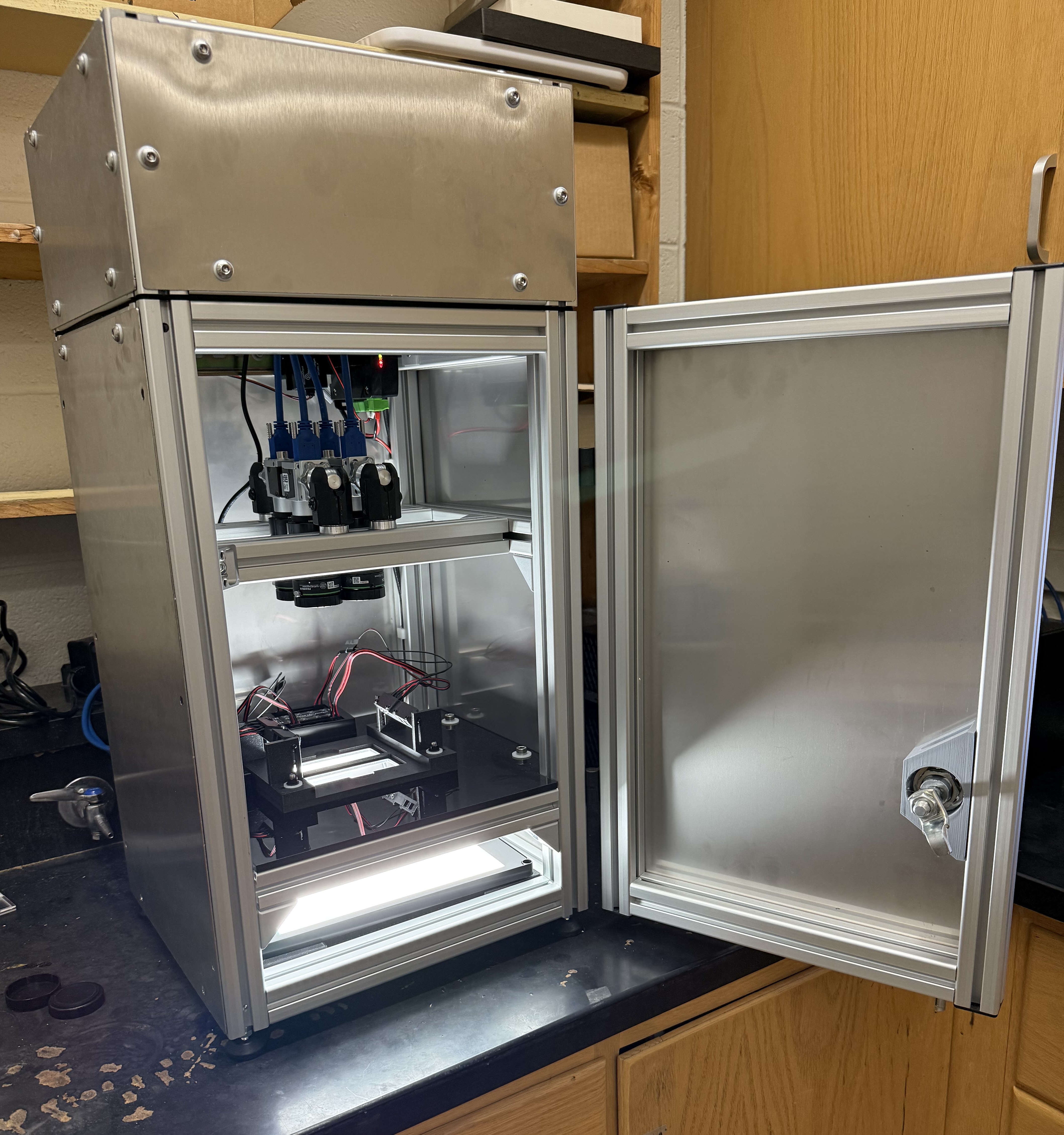

Supplement: S1 Fig — (JPEG) [file ppat.1013766.s001.jpeg]

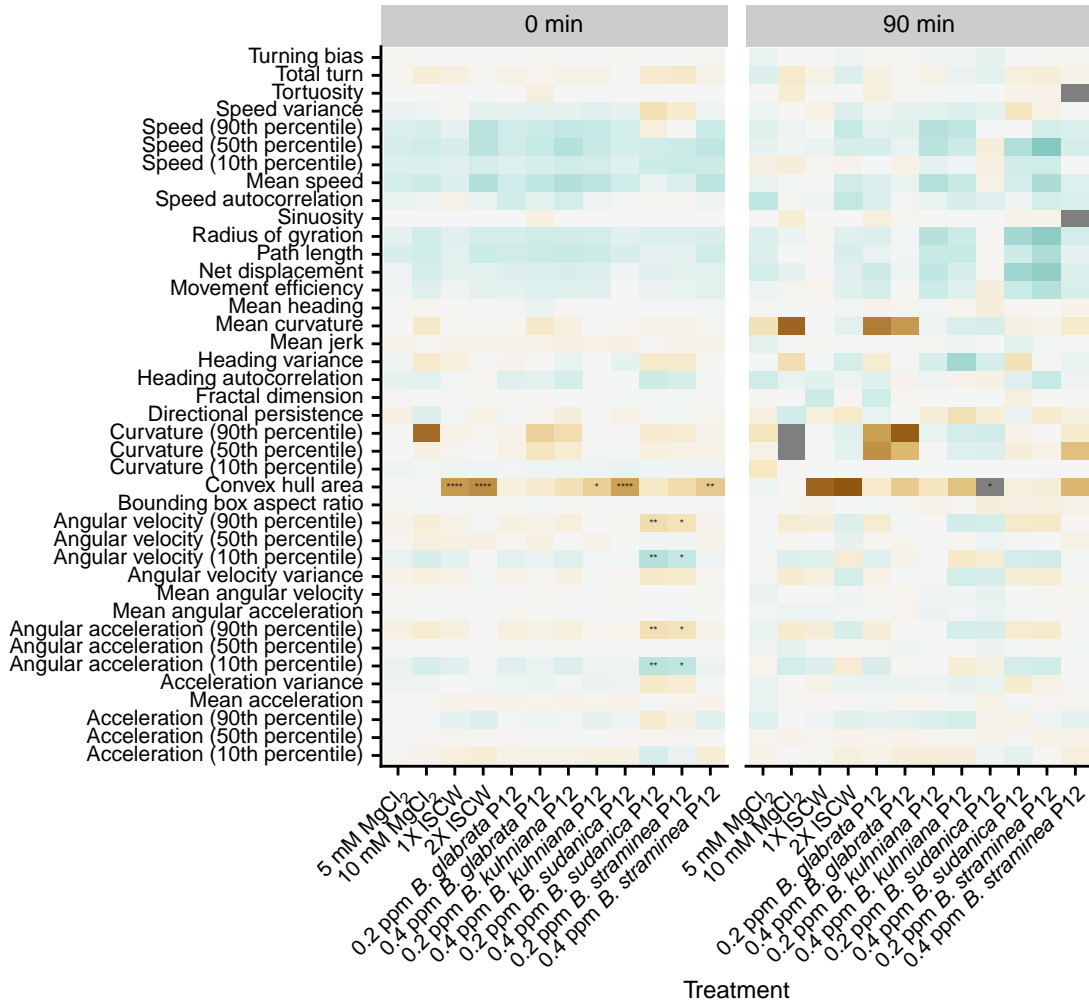

Standardized  
effect size

-1.0 -0.5 0.0 0.5 1.0

Supplement: S2 Fig — Data for 30 and 60 minutes is shown in Fig 4. (PDF) [file ppat.1013766.s002.pdf]

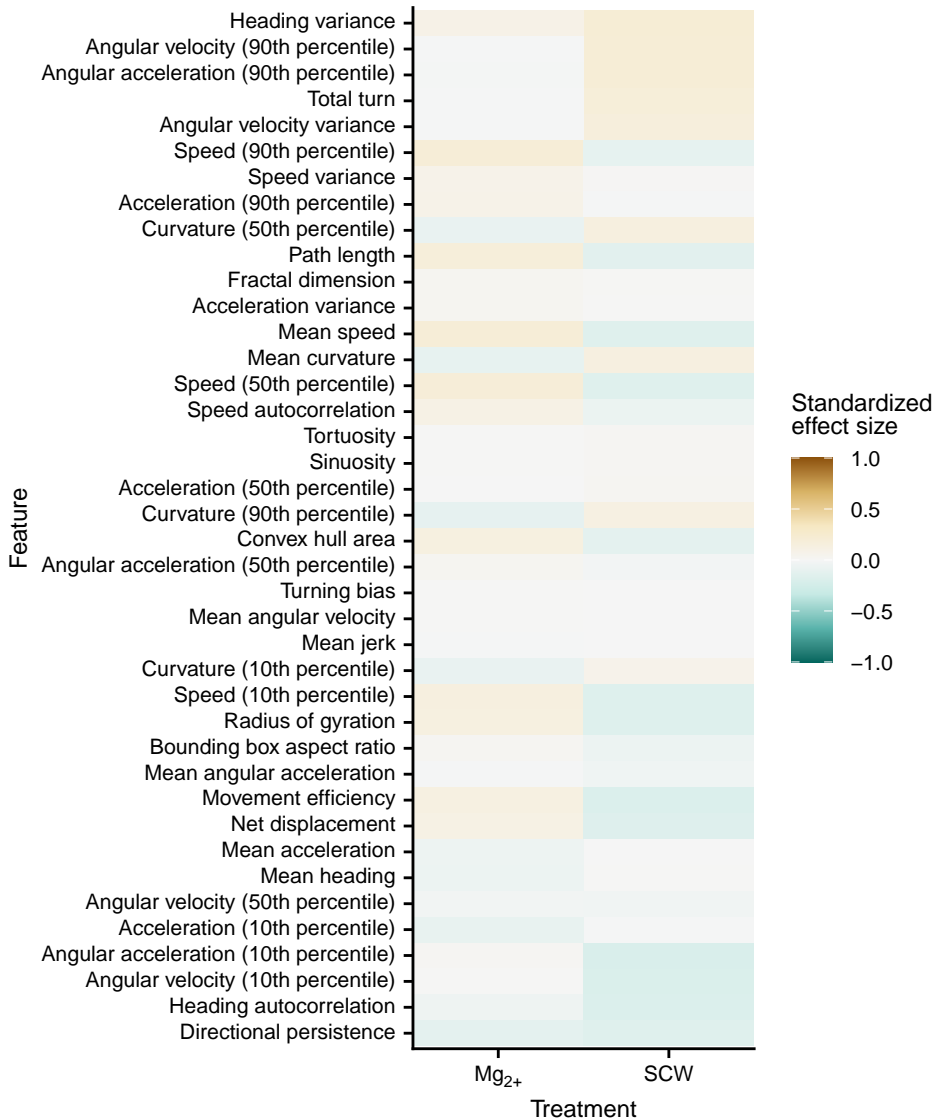

Supplement: S3 Fig — (PDF) [file ppat.1013766.s003.pdf]

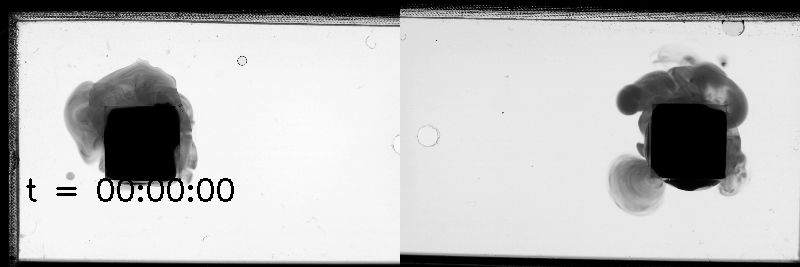

Supplement: S1 Movie — (GIF) [file ppat.1013766.s004.gif]

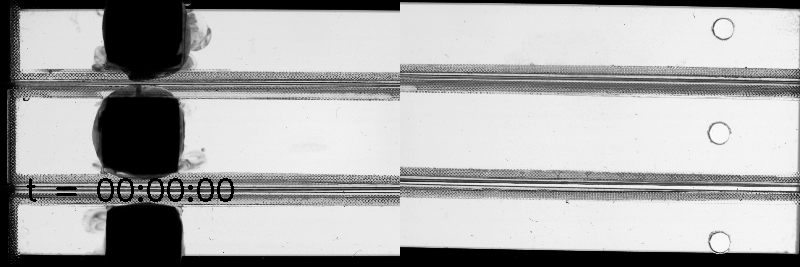

Supplement: S2 Movie — (GIF) [file ppat.1013766.s005.gif]
