# Supplementary material for: Quantitative ethology of schistosome miracidia characterizes a conserved snail peptide that inhibits host recognition
Source: PLoS Pathog. 2025 Dec 9;21(12):e1013766. doi: 10.1371/journal.ppat.1013766 (PMC12704881; doi:10.1371/journal.ppat.1013766)
Supplement: S1 Methods — (DOCX) [file ppat.1013766.s007.docx]

# Supplementary Methods

## InVision design

For high-resolution wide-field imaging, we designed the InVision (for invertebrate vision) by customizing a Kastl-HighRes unit controlled by the Motif software (Loopbio GmbH, Vienna, Austria) based on inspiration from similar recording devices for *C. elegans* (1). The InVision system includes four cameras (Basler acA5472-17um, Ahrensburg, Germany) and lenses (Fujifilm Fujinon CF50ZA-1S, Tokyo, Japan) affixed to T-slotted extrusion bars in an aluminum enclosure (S1 Fig). The enclosure is not environmentally controlled but has two exhaust fans to maintain ambient temperature. Arenas sit upon a laser-cut black acrylic stage with a 200 x 200 mm white, infrared (850 nm), or red (625 nm) LED panel (MBJ DBL-2020 series, Ahrensburg, Germany) below to generate a homogenous bright background by transillumination. The InVision also contains RGB LEDs on mounts to provide epi-illumination, but the functionality was not used for this project.

Cameras are paired to create two distinct fields of view (FoV) of 81x27 mm, with the FoV of paired cameras overlapping ~10%. The 1” camera sensors at 200 mm working distance creates an image with a resolution of 126.5 px/mm, meaning a miracidium consists of an ellipsoid ~25 pixels long and ~8 pixels wide. The Motif software synchronizes camera capture while recording frame-wise environmental variables via Phidget sensors (humidity, temperature, and luminosity). Motif utilizes GPUs to compress videos in real time according to the HEVC (H.265) standard and stores them in a MP4 container. HEVC efficiency relies upon a homogenous background, and our arena design (see below) routinely allowed compression ratios up to 100:1 without sacrificing tracking of extremely small animals. Videos are stored on a network attached storage device (Synology, New Taipei City, Taiwan) and transferred to the UWEC

## Arena and agarose mold design and fabrication

Design files (PDF, STL, or CAD) for arenas and molds can be found in GitHub repository associated with this manuscript (wheelerlab-uwec/miracidia-sensation-ms) or the archived release on Zenodo (10.5281/zenodo.15787645). All arena parts were fabricated from 1.6 mm (1/16”) thick clear or black cast acrylic (McMaster-Carr, Elmhurst, IL USA) laser-cut with a Trotec Speedy 360 80-Watt laser CO2 engraver/cutter (Marchtrenk, Austria). Parts were custom designed to perfectly fit the InVision stage. For choice and single-cue ethology arenas, three clear pieces were cut: a base with an engraved groove for seating the frame, a frame, and a top with engraved groove and cut inlets for loading agarose casts or parasites. For screening arenas, a single clear base was cut, and a black top was cut with wells designed to fit a multichannel pipette. Each well could fit 16 µL of liquid and avoid creating a meniscus, thus minimizing shadowing in videos. The top was also lightly engraved throughout to aid in adherence during and after fabrication. All arenas limit miracidia movement in the Z-axis, maintaining the parasites within the cameras’ focal plane.

Agarose molds were designed in OnShape (Boston, MA USA) and printed with PLA or PETG filament on Prusa MK3S or XL 3D printers (Prague, Czech Republic). Printers used a 0.4 mm nozzle with 0.2 mm layer height and 15% infill. Molds were designed to create casts that perfectly fit the ethology arenas and hold 180-200 µL of liquid.

Ethology and screening arenas were fabricated by a standard process. Following fabrication, all acrylic pieces were rinsed under tap water to remove residual dust from laser-cutting and then dried thoroughly. For ethology arenas, methyl ethyl ketone (MEK, an acrylic solvent) was applied to the contact surfaces using a micropipette. Approximately 50 µL of MEK was dispensed along each long edge of the frame and 10 µL along each short edge. The frame was aligned with the engraved groove on the base and pressed firmly by hand for several minutes to initiate solvent bonding. Once secure, the top layer was positioned and glued using the same MEK volumes, ensuring proper alignment of the agarose slots and miracidia inlet. Assembled ethology arenas were sealed with tape over the inlets to prevent dust contamination during storage.

For screening arenas, a thin, even layer of MEK was first applied to the bonding surfaces of the clear base and the engraved side of the black acrylic top using a fine paintbrush. Once aligned and pressed together, an additional 100 µL of MEK was slowly pipetted along the entire seam between the two layers to ensure complete bonding. The assembled screening arenas were clamped between two flat wooden boards for several minutes to apply even pressure. Once dry, they were covered with tape and stored in a dust-free container until use.

## P12 cloning, sequencing, and synthesis

Predicted sequences of BGLBO28940 and BGLBO27975 were obtained from the *B. glabrata* BBO2 reference genome (2), available on VectorBase (3), and Primer3 (4) was used to design PCR primers that would amplify the full-length mRNA and the coding sequence of both genes. The predicated protein sequence was used in tblastn search to identify potential homologs in other snails.

RNA was extracted from *B. glabrata* (NMRI), *B. sudanica* KEMRI, and *B. kuhniana* Grande Riviere (5) snails using the Direct-zol RNA Miniprep (Zymo Research, Irvine, CA USA). One *B. glabrata* snail, 1 cm in width, was placed in a glass petri dish, cleaned with 70% ethanol and wiped down using Kimwipes to remove contaminants. *B. sudanica* and *B. kuhniana* snails, obtained from collaborators at the University of New Mexico and stored in Trizol at -80°C, were thawed, removed and cleaned using the same procedure. A small beaker was firmly pressed on top of the snails to shatter the shell, and dissection tools were used to remove the snail tissue from its shell. The tissue was transferred to 1.5 mL RNase/DNase-free tubes and 400 µL of Trizol was added. Sterile plastic pestles were used to homogenize the tissue. The tube was capped, dropped in liquid nitrogen, removed after complete frozen, and homogenized again with the pestle. After homogenization, the tube was centrifuged at 21,300 rcf for 1 minute at room temperature. The supernatant was transferred to a spin column and the manufacturer’s protocol was followed with 10,000 rcf centrifugation steps.

A NanoDrop One (ThermoFisher, Waltham, MA USA) was used to evaluate nucleic acid purity and a Qubit4 fluorometer (ThermoFisher) was used to quantify the extracted RNA. The SuperScript IV First-Strand Synthesis System (ThermoFisher) was used to convert the RNA to cDNA. PCR was used to amplify P12 from the cDNA using the 2X Platinum SuperFi II PCR Master Mix (ThermoFisher). Six reactions were performed in total with the primers designed to amplify the full-length mRNA and the coding sequence for both genes (BGLBO28940 and BGLBO27975) from all three *Biomphalaria* species. Amplification was accomplished using the following cycling conditions: 98°C for 20 seconds, followed by 35 cycles of 98°C for 10 seconds, 60°C for 10 seconds, 72°C for 30 seconds, and finished with 1 cycle at 72°C for 5 minutes and held at 4°C.

Amplified cDNA products were cleaned using the DNA Clean & Concentrator (Zymo). The manufacturer protocol was followed exactly with a 5:1 DNA Binding Buffer to sample ratio. Centrifugation was performed at 10,000 rcf for 30 seconds at room temperature for all steps. Samples were sent to Plasmidsaurus (South San Francisco, CA USA) for amplicon sequencing. A multiple sequencing alignment (MSA) was performed with the sequences from *B. glabrata*, *B. kuhniana*, and *B. sudanica*, and the sequences of *B. straminea* (6) and *B. pfeifferi* (7) using AliView software (8). Four unique versions of P12 were identified from the MSA, and each version was synthesized for use in ethology assays and host recognition experiments (GenScript, Piscataway, New Jersey).

Amplified cDNA products were cloned into pCR-Blunt II-TOPO plasmid vectors using the Zero Blunt TOPO PCR Cloning Kit (ThermoFisher) and transformed into One Shot TOP10 Chemically Competent *E. coli* cells. Steps from the manufacturer’s protocol were followed exactly. The cells were plated on LB + kanamycin (50 µg/mL) agar plates using sterile plating beads and incubated overnight at 37°C. Transformants were picked using sterile pipette tips and placed in 10 mL of LB + kanamycin (50 µg/mL) broth to culture overnight at 37°C in a horizontal test tube shaker set to 180 rpm.

Plasmids of P12 clones were purified using the ZymoPURE Plasmid Prep Kit (Zymo). Steps from the manufacturer protocol were followed exactly. The samples were sent to Plasmidsaurus for sequencing. Sequenced data was uploaded to GenBank (PV848035-PV848039).

1. Barlow IL, Feriani L, Minga E, McDermott-Rouse A, O’Brien TJ, Liu Z, et al. Megapixel camera arrays enable high-resolution animal tracking in multiwell plates. Commun Biol. 2022 Mar 23;5(1):1–13.

2. Adema CM, Hillier LW, Jones CS, Loker ES, Knight M, Minx P, et al. Whole genome analysis of a schistosomiasis-transmitting freshwater snail. Nat Commun. 2017 May 16;8(1):15451.

3. Giraldo-Calderón GI, Emrich SJ, MacCallum RM, Maslen G, Dialynas E, Topalis P, et al. VectorBase: an updated bioinformatics resource for invertebrate vectors and other organisms related with human diseases. Nucleic Acids Research. 2015 Jan 28;43(D1):D707–13.

4. Untergasser A, Cutcutache I, Koressaar T, Ye J, Faircloth BC, Remm M, et al. Primer3—new capabilities and interfaces. Nucleic Acids Res. 2012 Aug;40(15):e115.

5. Mukaratirwa S, Laidemitt MR, Hewitt R, Sengupta ME, Marchi S, Polius C, et al. Update on the Geographic Distribution of the Intermediate Host Snails of Schistosoma mansoni on St. Lucia: A Step Toward Confirming the Interruption of Transmission of Human Schistosomiasis. Am J Trop Med Hyg. 2023 Oct;109(4):811–9.

6. Nong W, Yu Y, Aase-Remedios ME, Xie Y, So WL, Li Y, et al. Genome of the ramshorn snail Biomphalaria straminea-an obligate intermediate host of schistosomiasis. Gigascience. 2022 Feb 15;11:giac012.

7. Bu L, Lu L, Laidemitt MR, Zhang SM, Mutuku M, Mkoji G, et al. A genome sequence for Biomphalaria pfeifferi, the major vector snail for the human-infecting parasite Schistosoma mansoni. PLoS Negl Trop Dis. 2023 Mar;17(3):e0011208.

8. AliView: a fast and lightweight alignment viewer and editor for large datasets | Bioinformatics | Oxford Academic [Internet]. [cited 2025 Mar 31]. Available from: https://academic.oup.com/bioinformatics/article/30/22/3276/2391211
